# Response of Soil Fungal Diversity and Community Composition to Varying Levels of Bamboo Biochar in Red Soils

**DOI:** 10.3390/microorganisms9071385

**Published:** 2021-06-25

**Authors:** Muhammad Waqqas Khan Tarin, Lili Fan, Dejin Xie, Muhammad Tayyab, Jundong Rong, Lingyan Chen, Muhammad Atif Muneer, Yushan Zheng

**Affiliations:** 1 College of Landscape Architecture, Fujian Agriculture and Forestry University, Fuzhou 350002, China; waqas_tarin@yahoo.com (M.W.K.T.); fafucly@fafu.edu.cn (L.C.); 2 College of Forestry, Fujian Agriculture and Forestry University, Fuzhou 350002, China; 2180428002@fafu.edu.cn (L.F.); 2160482002@fafu.edu.cn (D.X.); rongjd@fafu.edu.cn (J.R.); 3 College of Agriculture, Fujian Agriculture and Forestry University, Fuzhou 350002, China; tyb.pk@hotmail.com; 4 International Magnesium Institute, College of Resources and Environment, Fujian Agriculture and Forestry University, Fuzhou 350002, China; m_atifmuneer@yahoo.com

**Keywords:** bamboo biochar, ecological functioning, fungal communities, forest management, soil characteristics

## Abstract

Soil fungi play a vital role in soil nutrient dynamics, but knowledge of their diversity and community composition in response to biochar addition into red soil is either limited or inconsistent. Therefore, we determined the impact of bamboo biochar (BB) with increasing concentrations (0, 5, 20, and 80 g kg^−1^ of soil, referred to as B0, BB5, BB20, and BB80, respectively) on soil physicochemical properties and fungal communities (Illumina high-throughput sequencing) in red soil under *Fokenia hodginsii* (Fujian cypress). We found that increasing BB levels effectively raised the soil pH and soil nutrients, particularly under BB80. BB addition significantly increased the relative abundance of important genera, i.e., *Basidiomycota*, *Mucoromycota,* and *Chytridiomycota* that could play a key role in ecological functioning, e.g., wood degradation and litter decomposition, improvement in plant nutrients uptake, and resistance to several abiotic stress factors. Soil amended with BB exhibited a substantial ability to increase the fungal richness and diversity; BB80 > BB20 > BB5 > B0. *Basidiomycota*, *Mucoromycota*, *Glomeromycota*, *Rozellomycota*, *Aphelidiomycota*, *Kickxellomycota,* and *Planctomycetes* were positively associated with soil pH, total nitrogen, phosphorous, and carbon, and available potassium and phosphorous. Besides, the correlation analysis between the soil fungal communities and soil properties also showed that soil pH was the most influential factor in shaping the soil fungal communities in the red soil. These findings have significant implications for a comprehensive understanding of how to ameliorate acidic soils with BB addition, as well as for future research on sustainable forest management, which might increase soil fungi richness, diversity, and functionality in acidic soils.

## 1. Introduction

Soil acidification is a key problem for terrestrial ecosystems and forest productivity [1]. Globally, 30% of the total land is comprised of acidic soils [2,3], and anthropogenic activities like intensive or inappropriate fertilization result in severe problems of soil acidification [4,5]. Soil acidification has been the biggest challenge to Chinese intensive farming systems since 1980, and in turn, modifying the soil physicochemical properties with negative effects on soil microbiota [6,7]. Therefore, the use of innovative technology to ameliorate soil acidification is of global concern for maximizing forest sustainability. 

The application of inorganic fertilizers is adequate to ensure forest productivity and plant growth [8]. The organic amendments from various sources, including forestry and agriculture, and urban areas, combined with inorganic fertilizer, are one appropriate way to alleviate such problems of soil acidification triggered by inorganic fertilizer. Previous research has shown that organic additions into the soil from diverse sources may enhance soil physicochemical and biological properties [9,10]. Recently, the addition of biochar to alleviate soil acidification has received a lot of interest across the globe. Biochar is black carbon that is processed by thermal degradation of organic substances under zero or limited oxygen (pyrolysis) [11]. Its influence on soil quality has been demonstrated primarily by raising soil pH in acidic soils [12], increasing nutrient retention by cation adsorption, or may shift soil microbial community composition and abundance [11]. Such changes might bring benefits to nutrient cycling, soil structure, and indirectly affect plant growth [13]. Therefore, potential interactions between soil physicochemical properties amended with biochar and soil microbes need to be further elucidated.

The soil amendment with biochar has a substantial effect on biotic and abiotic soil properties, which could effectively change the soil microbial diversity, community composition, and abundance [14]. Chen et al. [15] stated that the biochar addition to the organic carbon-poor dry soil enhanced the soil microbial diversity and abundance. Liu et al. [16] also reported that biochar substantially improved soil fertility and yield by increasing the relative abundance of soil fungi and bacteria and changed the community structure. A large number of experiments have focused on the microbial effects of biochar and chemical fertilizers, but most of the reports devoted more attention to bacteria [17,18,19]. Since fungi are the primary decomposer and carbon sequester in the forest ecosystem, and their role in the conservation of soil fertility and health is critical. 

Fungal communities, in addition to having a substantial influence on soil health and plant growth, are likely to survive under adverse environmental conditions and important biological components that trigger a variety of ecological functions, such as organic matter decomposition, parasitism, and controlling of the soil nutrient [20,21,22]. For instance, in a Tibetan forest, changing soil pH influenced the fungal alpha diversity [23], and this has not always been the fact when concerning fungal diversity. For example, Rousk et al. [24] reported that, although fungal diversity was correlated with soil pH, the relationship was considerably weaker relative to that of soil pH and bacterial diversity. In addition, the soil organic carbon and plant species have been found to be the key determinants in regulating fungal diversity in soils on China’s Loess Plateau and grassland soils of the Tibetan plateau [25,26]. Besides, various nutrients, such as organic carbon [27], available phosphorus [28], and various nitrogen forms [29], also greatly impact fungal diversity. There is a complex network of soil fungi, and their distribution and nature fluctuate in space [30]. Although the importance of bacterial community composition and their interaction with environmental factors has recently been substantially investigated, soil fungal communities are not well known as soil bacteria, despite their great biodiversity and crucial relevance in ecological functioning.

Evidence suggests that the biochar application rate, its properties, or the production conditions are the key factors influencing the fungal communities and the supply of nutrients for the sustainable management of agricultural ecosystems [31]. As a result, the concern of how biochar impacts fungal community composition in red soil under coniferous species is of increasing interest. *Fokenia hodginsii* (*F. hodginsii*) is a valuable tree species native to southern China, Vietnam, and Laos and has gained a great deal of interest in China due to its high-quality timber. In our previous research, we observed that different concentrations of bamboo biochar (BB) increased the biomass and root morphological features of *F. hodginsii* due to improved soil properties [32,33]. Therefore, we further investigated the impact of different concentrations of BB on soil characteristics, as well as the structure and diversity of fungal communities in red soils. We hypothesized that differences in fungal communities due to the application of BB at different concentrations may alter the abundance of various fungal taxa. The study objectives were; (1) to evaluate the effects of various BB concentrations on fungal diversity and community composition in red soil and (2) to determine the key environmental factors that shape the fungal diversity and community composition.

## 2. Materials and Methods

### 2.1. Experimental Setup

The current study was carried out in Bamboo Institute of the Fujian Agriculture and Forestry, Fuzhou, Fujian, China. One-year-old *F. hodginsii* seedlings of similar growth were grown in red soil amended with BB. Prior to BB integration, the basic physicochemical properties of both the soil and the biochar were assessed and have been presented in Table S1. Red soil was mixed with four different concentrations of BB, i.e., 0, 5, 20, and 80 g kg^−1^ of soil, named B0, BB5, BB20, and BB80, respectively. We planted one seedling in each polyvinyl pot (height = 18 cm, diameter = 22 cm top circumference = 62 cm, bottom circumference = 52 cm, and soil weight = 5 kg of soil per pot) and established 24 seedlings in total. However, we selected three pots from each treatment to assess the soil physicochemical properties and DNA extraction. All replicates were cultivated in a glasshouse with adequate irrigation and natural light to produce healthy seedlings and were arranged in a completely randomized design. In addition, after 15 days of the establishment, we applied 10 g of compound fertilizer (granular: NPK, 15:15:15) to each pot [34] as recommended by the Anxi Forest Nursery. After one year, the seedlings were harvested and the uprooted seedlings were gently shaken to extract rhizosphere soil. The soil samples were stored immediately in a sterile icebox and transported to the laboratory. All soil samples were sieved and separated into two subsamples; one was air-dried to estimate soil physicochemical properties and the other was stored at −80 °C for the DNA extraction.

### 2.2. Determination of Soil Physicochemical Properties

To assess the soil physicochemical properties, initially, soil samples were air-dried and sieved (0.149 or 2 mm). The soil pH (1:2.5 soil/water suspensions) was determined using a glass electrode meter (Seven Compact; Mettler-Toledo, Greifensee, Switzerland) [35]. Soil total carbon (TC) and total nitrogen (TN) were measured using the Elemental Analyzer (Thermo Scientific^TM^, Waltham, MA, USA). Total phosphorus (TP) and available phosphorus (AP) were estimated using the alkali fusion-Mo-Sb Anti-colorimetric method [36] on a spectrophotometer (BioTek, Epoch2, Winooski, VT, USA) at an absorbance wavelength of 700 nm. For the determination of available potassium (AK), the ammonium acetate solution was used and then measured at a flame photometer (FP640^®^, AOPU Analytical Instruments, Shanghai, China) [37].

### 2.3. DNA Extraction and PCR Amplification

Total genomic DNA was extracted from soil samples using the Fast DNATM Spin kit (MP Biomedical, Santa Ana, CA, USA); following manual instructions, DNA was purified with a DNA purification kit (Tiangen Biotech Co., Ltd., Beijing, China). The quality and quantity of DNA were estimated using NanoDrop (Thermo Fisher Scientific, Middletown, VA, USA) and later preserved at −20 °C for sequencing. ITS2-110 2043R and ITS5-1737F primers were used to amplify the ITS1 fungal region [38]. The PCR reactions were performed in 30-μL mixtures for each primer (0.2 μM); Phusion^®^ High-Fidelity PCR Master Mix (15 μL) (New England BioLabs, Ipswich, MA, USA) and DNA templates (10 ng). The conditions set for the PCRs were 98 °C~one-min, following 30 Cycles of 98 °C~10 s, 50 °C~30 s, 72 °C~60 s, and with a final extension of 72 °C~5 min. QIAquik Gel Extraction Kit (QIAGEN, Düsseldorf, Germany) was used to purify the PCR products. TruSeq^®^ DNA PCR-Free Sample Preparation Kit (Illumina, San Diego, CA, USA) was used to develop sequencing libraries. Whereas, Qubit @ 2.0 fluorometer (Thermo Fisher Scientific, Waltham, MA, USA) and the Agilent Bioanalyzer 2100 system (Santa Clara, CA, USA) were used for their quantity measurements. At last, the DNA libraries were sequenced by Novogen (Beijing, China) on the Illumina HISeq2500 platform. All the sequencing data were deposited in a NCBI SRA database with accession number PRJNA 735056.

### 2.4. Statistical and Bioinformatic Analyses

Using FLASH (Baltimore, MD, USA), the paired end reads from the initial DNA fragment were combined based on the unique barcode assigned to each sample. The sequences were assigned to the operational taxonomic unit (OTU) based on 97% of similarity. Representative sequences were chosen for each OTU, and taxonomic information was annotated for each representative sequence using a ribosomal database project (RDP) classifier [39]. The alpha diversity and species richness were quantified using the Shannon, Simpson, Chao1, and ACE indices [40,41,42]. The rarefaction curves were generated based on the observed species richness, and the Venn diagram displayed the unique and common OTUs among the soil samples. In addition, unweighted UniFrac principal coordinate analysis (PCoA), unweighted UniFrac pair group approach with arithmetic means analysis (UPGMA), and analysis of similarities (ANOSIM) were also employed to investigate variations in species complexity between samples. A redundancy analysis (RDA) was performed to analyze the relationship between the fungal community structure at the phylum and genus level and soil physicochemical characteristics. Pearson correlation analysis between soil properties and soil fungi diversity, phyla, and genera were also carried out. The least significant difference (LSD) test was performed to determine the significant differences in soil physicochemical properties. For visualization, we used R software (version 2.15.3, R Foundation for Statistical Computing, Vienna, Austria) and Origin^®^ v. 8.5 (Origin-Lab Corp., Northampton, MS, USA).

## 3. Results

### 3.1. Effect of Bamboo Biochar on Soil Physicochemical Characteristics 

**Figure 1:**
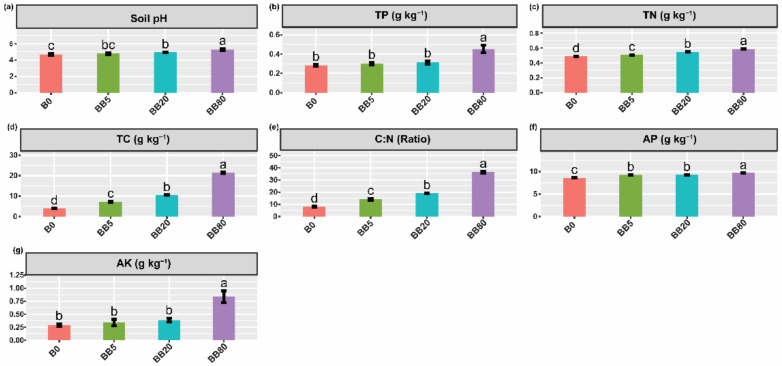
The effects of different concentration of bamboo biochar on soil physicochemical properties. (**a**) Soil pH, (**b**) Total phosphorous (TP); (**c**) Total nitrogen (TN); (**d**) Total carbon (TC); (**e**) C:N ratio; (**f**) Available phosphorous (AP); (**g**) Available potassium (AK). The bar graphs with different lowercase letters show a significant difference between treatments (LSD test, *p* < 0.05). B0, BB5, BB20, and BB80 represent different bamboo biochar concentrations of 0, 5, 20, and 80 g kg^−1^, respectively.

The application of BB had a significant effect on soil physicochemical properties (Figure 1). We found that increasing biochar concentration effectively increased the soil pH and soil nutrient levels, e.g., TP, TN, TC, C:N, AP, and AK. Collectively, it implies that the biochar amendment not only alleviated soil acidification but also improved the soil nutrient status. Specifically, BB80 resulted in a considerable increase in soil nutrient contents relative to all other treatment combinations (Figure 1).

### 3.2. Effect of Bamboo Biochar on Soil Fungal Community

**Figure 2:**
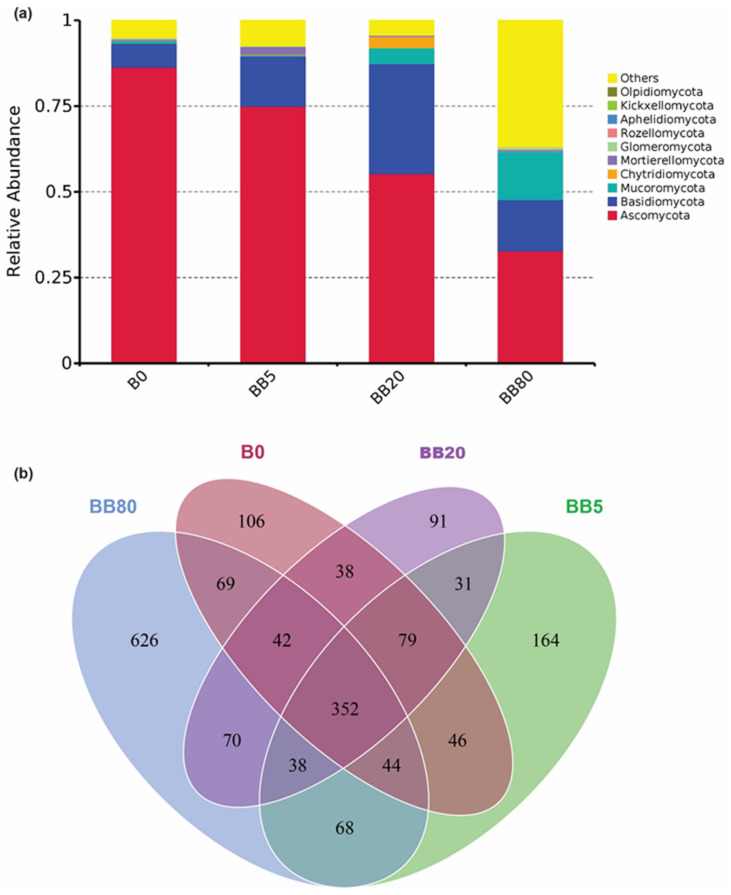
Relative abundance of topsoil fungal species. (**a**) The relative abundance of topsoil fungal communities at Phylum level; (**b**) Comparison of different soil fungal communities under different treatments of bamboo biochar. The relative abundance of the top 10 phyla has been shown and unclassified/less abundant classified as others. B0, BB5, BB20, and BB80 represent different bamboo biochar concentrations of 0, 5, 20, and 80 g kg^−1^, respectively.

The relative abundance of fungal phyla changed with BB concentration (B0, BB5, BB20, and BB80). The *Ascomycota*, *Basidiomycota*, *Mucoromycota,* and *Chytridiomycota* were dominant phyla found in all treatments. The abundance of *Ascomycota* decreased significantly with increasing biochar concentrations, while an opposite response was observed for *Mucoromycota*. We also found that the relative abundance of *Basidiomycota*, *Chytridiomycota* was higher under BB treatments compared with control (B0) (Figure 2a). Overall, these results showed that BB contributed to improving the relative abundance in the red soil of southern China. Moreover, the Ven diagram also revealed that 352 OTUs were common, suggesting a higher similarity of soil fungal communities among all treatments. However, under the highest BB concentrations (BB80), the maximum number of unique OTUs (i.e., 626) were observed (Figure 2b).

### 3.3. Fungal Richness and Diversity Increased under Bamboo Biochar Amendments

**Figure 3:**
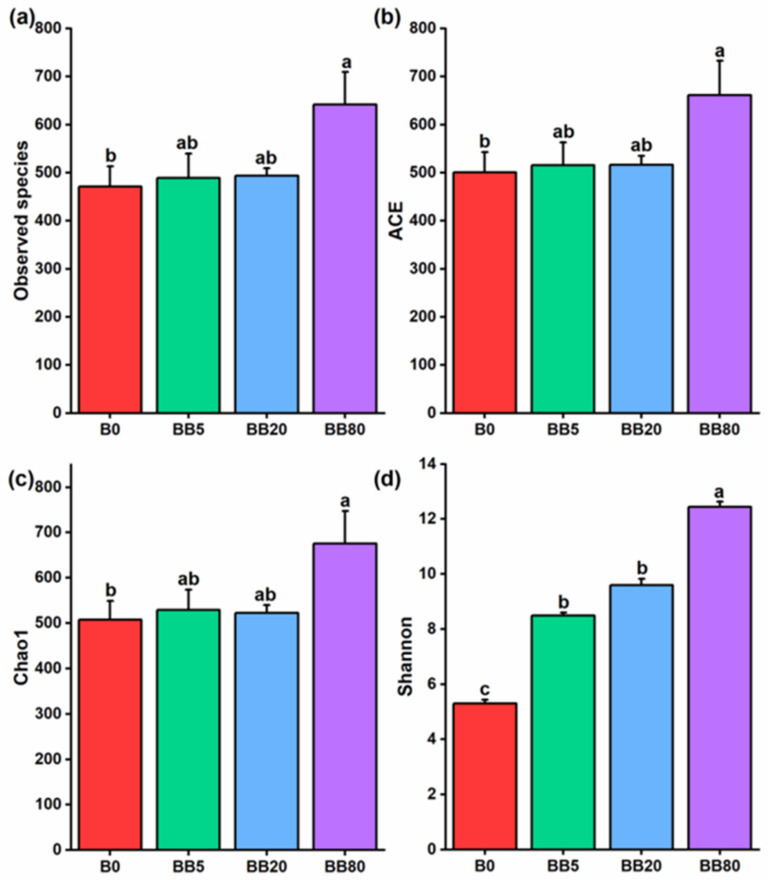
The alpha diversity of soil fungal species. The different alpha diversity indices were calculated under different bamboo biochar concentrations: (**a**) Observed species; (**b**) ACE index; (**c**) Chao1 index; (**d**) Shannon diversity index. The bar graphs with different lowercase letters show significant differences between various treatments (LSD test, *p* < 0.05). B0, BB5, BB20, and BB80 represent different bamboo biochar concentrations of 0, 5, 20, and 80 g kg^−1^, respectively.

To assess the impact of BB on fungal richness and diversity, four alpha diversity indices were investigated, including observed species, ACE, Chao1, and Shannon (Figure 3). We found that relative to B0, BB5 and BB20 did not show significant differences in soil fungal richness and diversity indices (observed species, ACE, and Chao1) (Figure 3a–c). However, all four indices (observed species, ACE, Chao1, and Shannon) were significantly higher, particularly under BB80 treatments relative to B0. In addition, the BB had a substantial ability to increase the Shannon index; BB80 > BB20 > BB5 > B0, with the highest alpha diversity under BB80 (Figure 3d). 

### 3.4. Changes in Soil Fungal Communities under Bamboo Biochar Concentration

**Figure 4:**
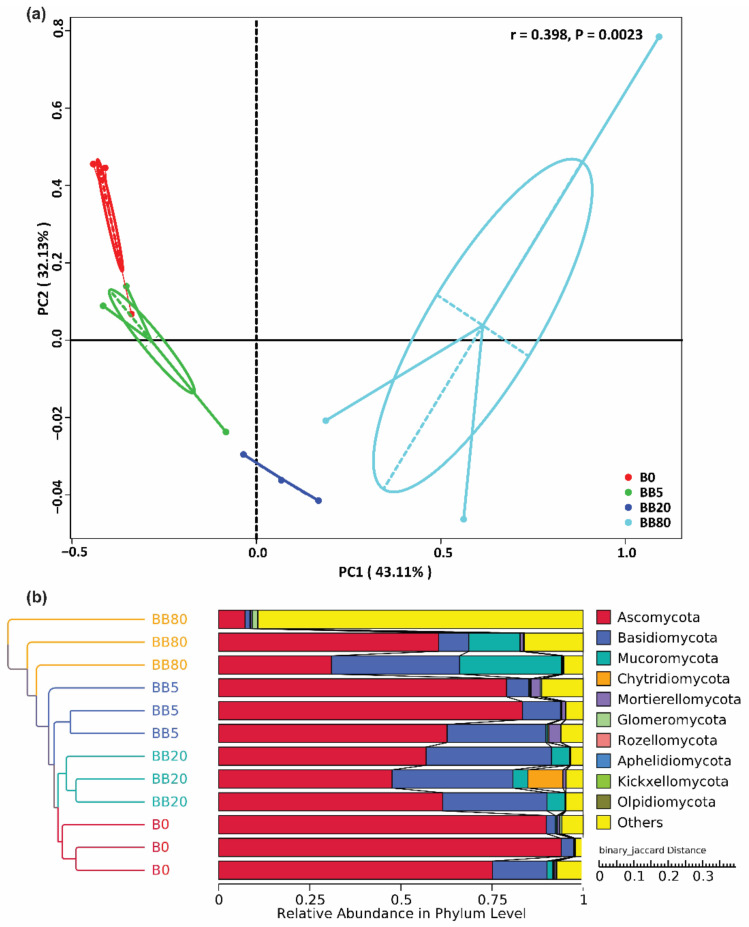
Changes in soil fungal community composition. (**a**) Unweighted UniFrac principal coordinate analysis (PCoA) showing differences in fungal communities; (**b**) Unweighted pair group method with arithmetic mean analysis (UPGMA) of fungal communities. B0, BB5, BB20, and BB80 represent different bamboo biochar concentrations of 0, 5, 20, and 80 g kg^−1^, respectively.

To evaluate whether either BB had a significant effect on soil fungal communities in comparison with no BB treatment, principal coordinate analysis was performed. The results showed the distinct patterns of fungal communities in response to varying BB concentrations, with the first and second axes representing a complete change of 75.24% in the fungal communities (Figure 4a). The unweighted pair group method with arithmetic mean analysis (UPGMA) further confirmed that the samples with varying biochar concentrations were well separated (Figure 4b), which was consistent with the analysis of similarities (ANOSIM) results (r = 0.398, *p* = 0.002), indicating that varying biochar concentrations strongly changed the soil fungal communities. Hence, we found that soil fungal communities were different from each other at different concentrations of BB.

### 3.5. Soil Properties Correlated with Soil Fungal Communities

**Figure 5:**
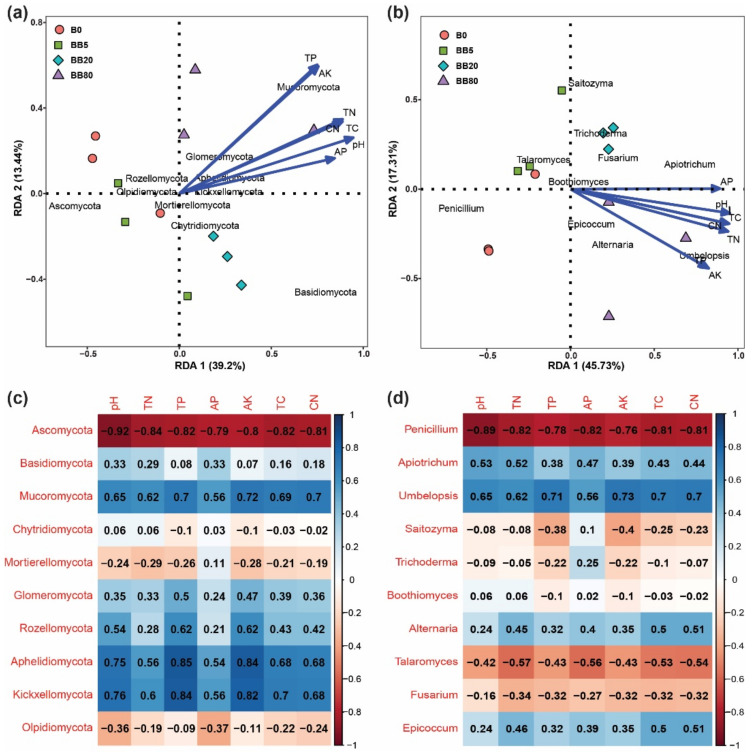
Effects of soil physicochemical properties on fungal communities. The effects of soil properties were tested on soil fungal communities: (**a**) RDA analysis (distance-based redundancy analysis) on phylum level; (**b**) Correlation analysis between soil properties and abundant taxa of fungi at phylum level; (**c**) RDA analysis on genera level; (**d**) Correlation analysis between soil properties and abundant taxa of fungi at the genera level.

The distance-based redundancy analysis (RDA) was performed to determine the environmental factors that affected fungal structure at the phylum and genus level. RDA results suggested that soil pH, TN, TP, AK, TC, AP, and C:N explained 52.64% and 63.04% of the total shift in fungal phyla and genera, respectively. Besides, under varying biochar concentrations, soil samples were completely separated from each other (Figure 5a,b). At the phylum level, *Ascomycota*, *Mortierellomycota*, and *Olpidiomycota* were negatively associated with soil pH, TN, TP, AK, TC, AP, and C:N, while *Basidiomycota*, *Mucoromycota*, *Glomeromycota*, *Rozellomycota*, *Aphelidiomycota*, *Kickxellomycota,* and *Planctomycetes* were positively associated (Figure 5c). Furthermore, at the genus level, *Penicillium*, *Saitozyma*, *Trichoderma*, *Boothiomyces*, *Talaromyces,* and *Fusarium* were negatively associated with soil pH, TN, TP, AK, TC, AP, and C:N, while *Apiotrichum*, *Umbelopsis*, *Alternaria,* and *Epicoccum* were positively associated (Figure 5d). Moreover, we also found that soil physicochemical properties including pH, TN, TP, AK, TC, AP, and C:N had a significant effect on soil fungal community composition (Table 1).

## 4. Discussion

In recent times, biochar is being practiced to raise the soil pH of acidic soil. Numerous studies have demonstrated that biochar has an excellent ability to improve soil properties owing to its unique biological and physicochemical properties, which induce shifts in soil microbial abundance and community composition. Therefore, we examined how varying BB concentrations affected soil characteristics as well as the fungal diversity and community composition in red soil with *F. hodginsii* plantation. As a result, our results may contribute to a better understanding of the impacts of varied BB concentrations on soil acidification, soil fertility, and soil fungal community composition, with an overall influence on soil health. 

We found that the BB amendment raised the soil pH and significantly improved the soil nutrient status (e.g., TP, TN, TC, C:N, AP, AK) (Figure 1). These findings are consistent with previous studies indicating that biochar not only helps to mitigate soil acidification but also improves the soil nutrient status [43,44]. This rise in soil pH is primarily due to BB high pH (Table S1) and a high concentration of base ions in its ash, such as Ca, Mg, K, and Na, which may efficiently decrease soil hydrogen ions and exchangeable aluminum ions [45], and therefore improves the soil nutrients status [46]. The better nutrient availability observed in this study could be related to direct input from biochar [11,47] because biochar itself does have the ability to improve soil fertility [48]. Thus, we concluded that the BB amendment to red soil significantly improved the soil physicochemical properties. 

Nevertheless, alleviating soil acidification and improving soil nutrient status in response to BB amendments may lead to an increase in the relative abundance of fungal species [49,50]. Therefore, we also found that relative abundance of *Basidiomycota, Mucoromycota*, and *Chytridiomycota* increased under BB (Figure 2). These results are similar to previous findings of Duan et al. [50] where the relative abundance of the soil fungal community also increased with increasing BB concentration. Generally, the relative abundance of *Ascomycota* was the highest under all treatments. *Ascomycota* is the most common and diversified phylum of eukaryotes, as well as the decomposition of organic substrate [22], and we discovered it to be the most common fungal phylum in the red soil. *Basidiomycota* includes some of the most well-known fungi for their ability to generate huge fruiting bodies, as well as plant parasite fungi that cause wood degradation and litter decomposition [51,52]. Because of their symbiotic relationship with the host plant’s roots, this fungus category could be very advantageous to plants, as they store mineral nutrients, metabolites, and water [52]. *Mucoromycota* fungal species can be found in a variety of habitats, and the majority of the AMF (arbuscular mycorrhizal fungi) species belong to *Mucoromycota* sub-phylum [53]. AMF are soil-borne fungi that can significantly improve plant nutrient uptake and resistance to several abiotic stress factors [54,55,56,57,58]. *Chytridiomycota* phylum diversity has been documented as a vital component in modern ecosystems that can live in a broad range of environments, as well as exist in temperature and moisture variations, and act as decomposers and bio-converters [59]. Hence, BB had a significant impact on the relative abundance of important fungal species for ecosystem functioning.

The alpha diversity indices revealed variations in soil fungal richness and diversity. The fungal species richness and diversity increased significantly with increasing biochar concentration (Figure 3) and were substantially affected by soil physicochemical properties (Figure 4). Soil nutrients and pH respond quickly to soil changes, so these are the most widely used indicators for the evaluation of soil microbial communities and to assess the soil quality [60]. In the current study, soil fungal richness and diversity increased, this could be due to improved soil physicochemical properties owing to the application of BB. The soil microbiota plays a vital role in soil function and ecosystem sustainability [61,62]. Therefore, studying the changes in soil microbial diversity under different BB concentrations can help to determine the possible reasons that result in the loss of soil microbial diversity, and it is considered a major threat to ecosystem functioning [63]. We also found that the soil fungal communities were significantly influenced by soil physicochemical properties (pH, TN, TP, AP, AK, TC, C:N ratio) (Figure 5, Table 1). We can infer that soil physicochemical properties changing with biochar concentration could contribute to the distinct variations in fungal community structures. Previous studies also showed that soil pH is the most important variable for shaping the fungal communities [64,65]. This is because soil pH may affect the fungal diversity and community composition by modifying nutrients availability or putting physiological limits on fungal growth. These findings complemented prior research that soil pH has a substantial impact on fungal populations [66,67]. The correlation analysis between the soil fungal communities and soil properties also showed that soil pH was the most influential factor affecting the soil fungal communities, and similar results have been reported in previous studies [68,69].

## 5. Conclusions

Overall, in this study, we investigated the response of soil fungal richness, diversity, and community composition to BB by Illumina high-throughput sequencing. We found that BB addition significantly increased the relative abundance of important genera, i.e., *Basidiomycota*, *Mucoromycota,* and *Chytridiomycota* that play key roles in ecological functioning, e.g., wood degradation and litter decomposition, improvement in plant nutrient uptake and resistance to several abiotic stress factors, etc. Moreover, both the soil fungal richness and diversity were significantly increased under BB80. The correlation analysis showed that soil pH was the most significant and influential factor in shaping the soil fungal communities in the red soil. Hence, we concluded that the addition of BB to the red soil had a significant effect in improving the soil physicochemical properties in terms of alleviating soil acidification. The improvement in soil physicochemical properties, especially the increase in soil pH, provided a suitable environment for soil fungal diversity and community composition. These findings have important implications for a comprehensive understanding of the improvement of acidic soils by the addition of BB, and also provide a synthesized insight for future studies on sustainable forest management that could improve the soil fungal richness, diversity, and functioning in acidic soils.

## Figures and Tables

**Table 1:** Pearson correlation (at phylum level) between the Bray-Curtis dissimilarity score and soil properties using the mantel test.

Variable Name	Corr-Method	Corr-Res	*p*-Res	Significance
pH	Pearson	0.711	0.004	**
TN	Pearson	0.871	0.001	***
TP	Pearson	0.650	0.013	*
AP	Pearson	0.779	0.004	**
AK	Pearson	0.667	0.007	**
TC	Pearson	0.862	0.001	***
C:N	Pearson	0.865	0.001	***

Level of significance at *p* < 0.05, *p* < 0.01, and *p* < 0.001 is denoted by *, **, and ***, respectively.

## Data Availability

All the sequencing data were deposited to NCBI SRA database with accession number PRJNA 735056.

## Supplementary Materials

The following are available online at https://www.mdpi.com/article/10.3390/microorganisms9071385/s1, Table S1: Basic properties of soil and biochar used in this study.
