# Supplementary material for: Response of Soil Fungal Diversity and Community Composition to Varying Levels of Bamboo Biochar in Red Soils
Source: Microorganisms. 2021 Jun 25;9(7):1385. doi: 10.3390/microorganisms9071385 (PMC8306102; doi:10.3390/microorganisms9071385)
Supplement: Supplementary file 1 [file microorganisms-09-01385-s001.zip › microorganisms-1272114-supplementary.pdf]

Table S1: Basic properties of soil and biochar used in this study.

Characteristics	Bamboo Biochar	Soil
pH	9.687	4.685
Electrical Conductivity ($mScm^{-1)}$	3.140	106.667
Total Nitrogen (g kg^{-1})	6.243	0.497
Total Carbon (g kg^{-1})	311.220	3.358
Total Phosphorous(g kg^{-1})	0.399	0.256
Available Phosphorous (mg kg^{-1})	32.381	10.245
Organic Matter (g kg^{-1})	536.543	5.790
Available Potassium (g kg^{-1})	0.335	0.356
C/N Ratio	49.975	6.864
